# Mechanisms underlying sex differences in autoimmunity

**DOI:** 10.1172/JCI180076

**Published:** 2024-09-17

**Authors:** DeLisa Fairweather, Danielle J. Beetler, Elizabeth J. McCabe, Scott M. Lieberman

**Affiliations:** 1 Department of Cardiovascular Medicine, Mayo Clinic, Jacksonville, Florida, USA.; 2 Center for Clinical and Translational Science, Mayo Clinic, Rochester, Minnesota, USA.; 3 Department of Immunology, Mayo Clinic, Jacksonville, Florida, USA.; 4 Division of Rheumatology, Allergy, and Immunology, Stead Family Department of Pediatrics, Carver College of Medicine, University of Iowa, Iowa City, Iowa, USA.

## Abstract

Autoimmune diseases are a leading cause of disability worldwide. Most autoimmune diseases occur more often in women than men, with rheumatic autoimmune diseases being among those most highly expressed in women. Several key factors, identified mainly in animal models and cell culture experiments, are important in increasing autoimmune disease in females. These include sex hormones, immune genes including those found on the X chromosome, sex-specific epigenetic effects on genes by estrogen and the environment, and regulation of genes and messenger RNA by microRNAs found in extracellular vesicles. Evidence is also emerging that viruses as well as drugs or toxins that damage mitochondria may contribute to increased levels of autoantibodies against nuclear and mitochondrial antigens, which are common in many autoimmune diseases. The purpose of this Review is to summarize our current understanding of mechanisms that may determine sex differences in autoimmune disease.

## Introduction

**Figure 1:**
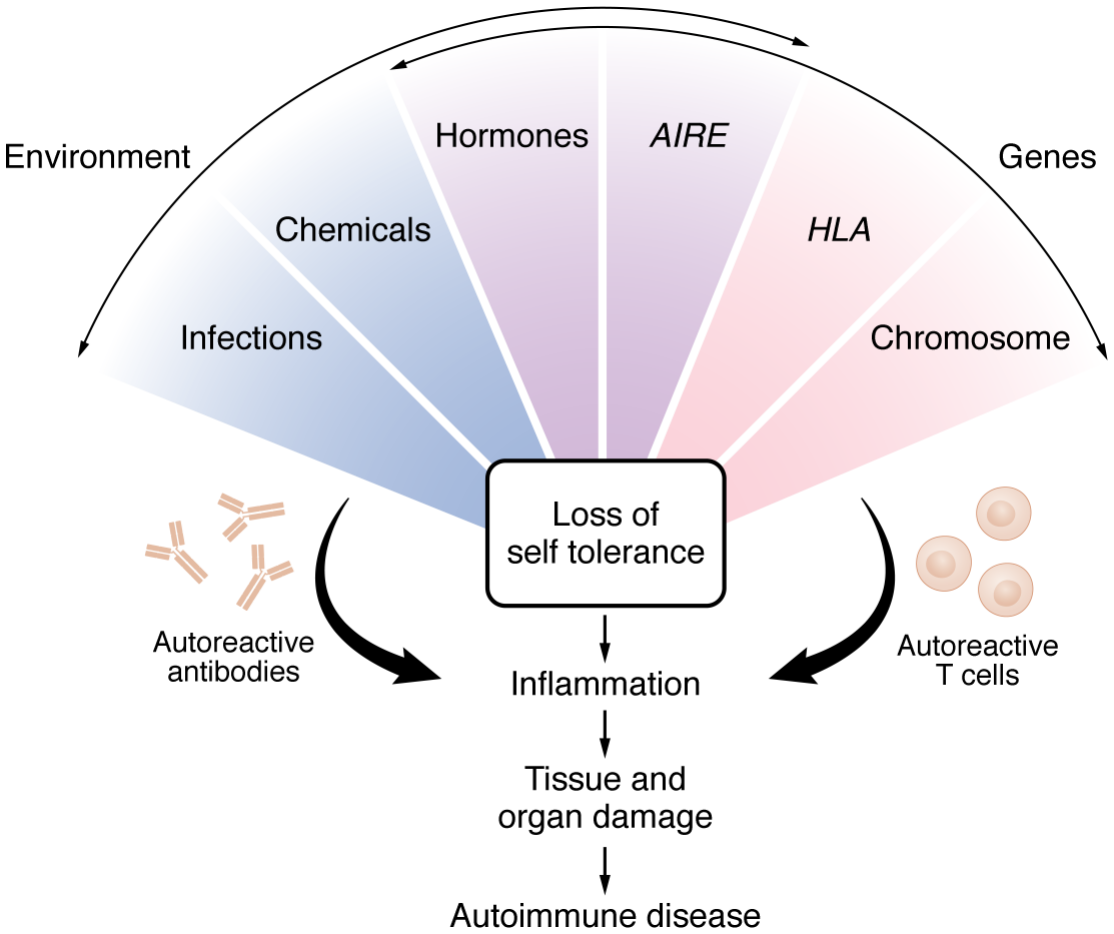
Mechanisms contributing to the development of autoimmune disease. A combination of genetic predisposition (Genes) and environmental factors (Environment) contributes to the development of autoimmune diseases. Genetic factors (red) include genes on the X chromosome that are not inactivated, such as FOXP3, which may lead to dysregulation of Tregs in females. HLA type is another example of a genetic factor that can increase susceptibility to developing an autoimmune disease. Environmental factors (blue) include chemicals and infections. Some genetic factors can be influenced by environmental factors (purple); for example, the autoimmune regulator gene (*AIRE*) can be decreased by viral infections or endocrine-disrupting chemicals may alter sex hormone signaling.

As a group, autoimmune diseases affect approximately 3%–10% of the population worldwide (1–5) and are the third most common category of disease in industrialized countries following cardiovascular disease and cancer. What constitutes an autoimmune disease depends on how they are defined (6), with the number of autoimmune diseases varying from 80 to 120 or more (7). In general, autoimmune disease develops when the innate and adaptive immune responses, which typically recognize and protect the host from invading pathogens or toxins, respond to self-antigens leading to loss of self-tolerance and chronic tissue damage (Figure 1). During maturation of the immune system, immune cells that react against self-antigens are eliminated in a process that is referred to as “central tolerance” (8). This protective process is incomplete and supplemented by several peripheral tolerance mechanisms, including the conversion of self-reactive Th cells to Tregs (9). A characteristic (and diagnostic) feature of autoimmune diseases is the presence of autoantibodies and autoreactive T cells (Figure 1) and a decrease in inhibitory Tregs (10, 11).

**Figure 2:**
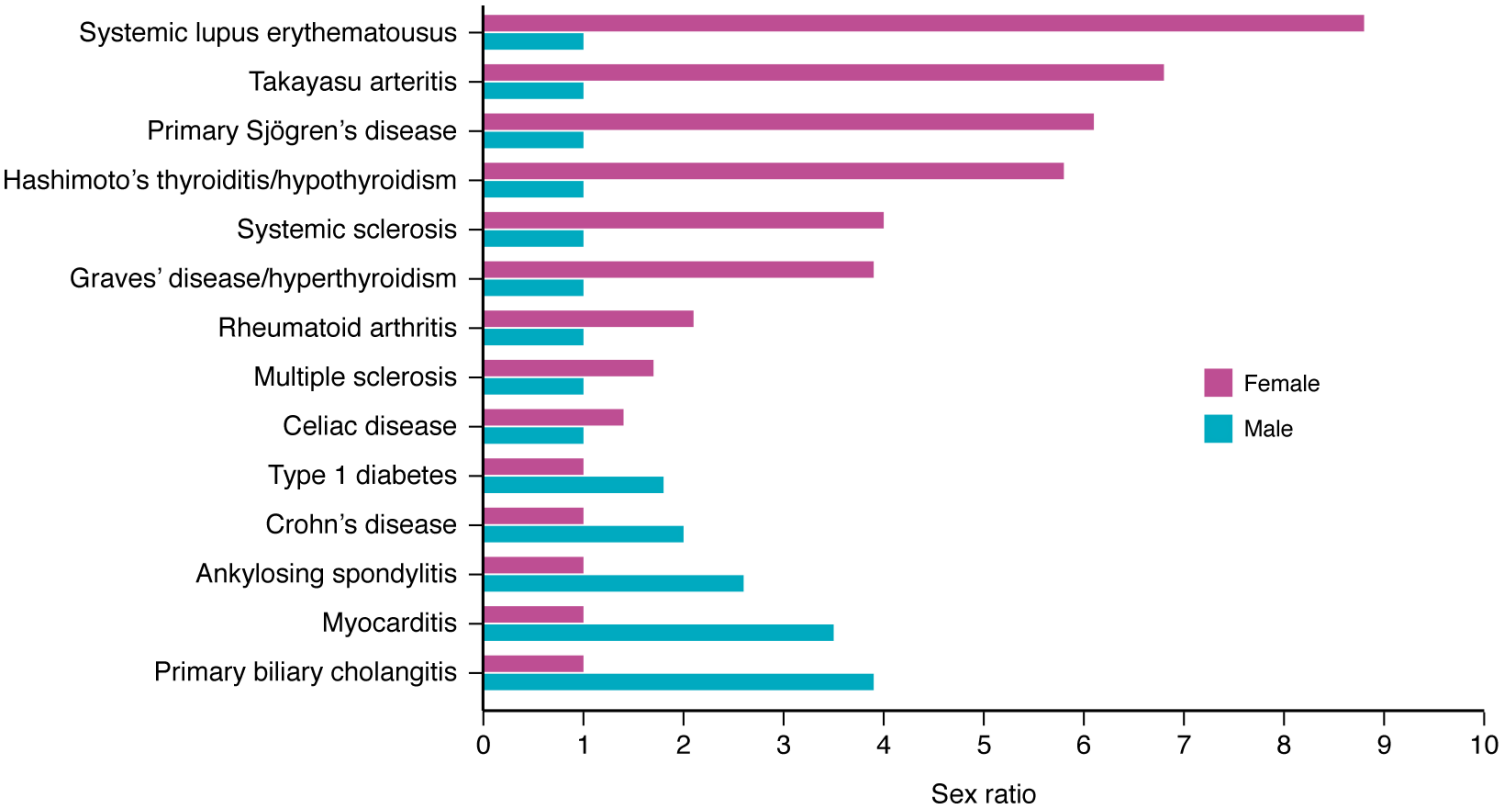
Sex differences in autoimmune disease. Most autoimmune diseases occur more often in women than men. Sex ratios comparing women with men for a number of autoimmune diseases are illustrated, including systemic lupus erythematosus (8.8:1) (12), Takayasu arteritis (6.8:1) (13), primary Sjögren’s disease (6.1:1) (15), thyroiditis (5.8:1) (16), systemic sclerosis (4:1) (167), Graves’ disease (3.9:1) (16), rheumatoid arthritis (2.1:1) (18), multiple sclerosis (1.7:1) (168), celiac disease (1.4:1) (169), type 1 diabetes (1:1.8) (170), Crohn’s disease (1:2) (171), ankylosing spondylitis (1:2.6) (172), myocarditis (1:3.5) (21), and primary biliary cholangitis (1:3.9) (20).

Most autoimmune diseases are more common in cisgender women (hereafter referred to as women) than cisgender men (hereafter referred to as men), especially autoimmune diseases that are more prevalent in the population. For example, sex ratios for autoimmune diseases that occur more often in women than men include systemic lupus erythematosus (SLE) (8.8:1) (12), Takayasu’s arteritis (6.8:1) (13), primary Sjögren’s disease (6.1:1) (14, 15), thyroiditis (5.8:1) (16), Graves’ disease (3.9:1) (17), rheumatoid arthritis (2.1:1) (18), and multiple sclerosis (1.7:1) (19) (Figure 2). However, some autoimmune diseases occur more often in men, such as primary biliary cholangitis (1:3.9) (20) and myocarditis (1:3.5) (21, 22) (Figure 2). There are several fundamental contributors to the sex differences in immune response that underlie autoimmune diseases, including sex hormones, genes, and environmental factors. The purpose of this Review is to summarize the current understanding of mechanisms that may determine sex differences in autoimmune disease.

## The role of sex hormones

Sex differences in systemic and tissue-specific inflammation are evident in most autoimmune diseases based on studies in humans and animal models (19, 23–26). The term sex refers to biological differences between males and females in, for example, anatomy, physiology, chromosomes, and genes, while gender refers to socially constructed roles, characteristics, and behaviors of men and women (27, 28). Very little data currently exist for other gender identities, which are understudied and so are not discussed here. This Review focuses on biological sex differences while acknowledging that sex and gender are intertwined, and both critically affect disease pathogenesis and outcome.

### Sex hormones and autoantibodies.

**Figure 3:**
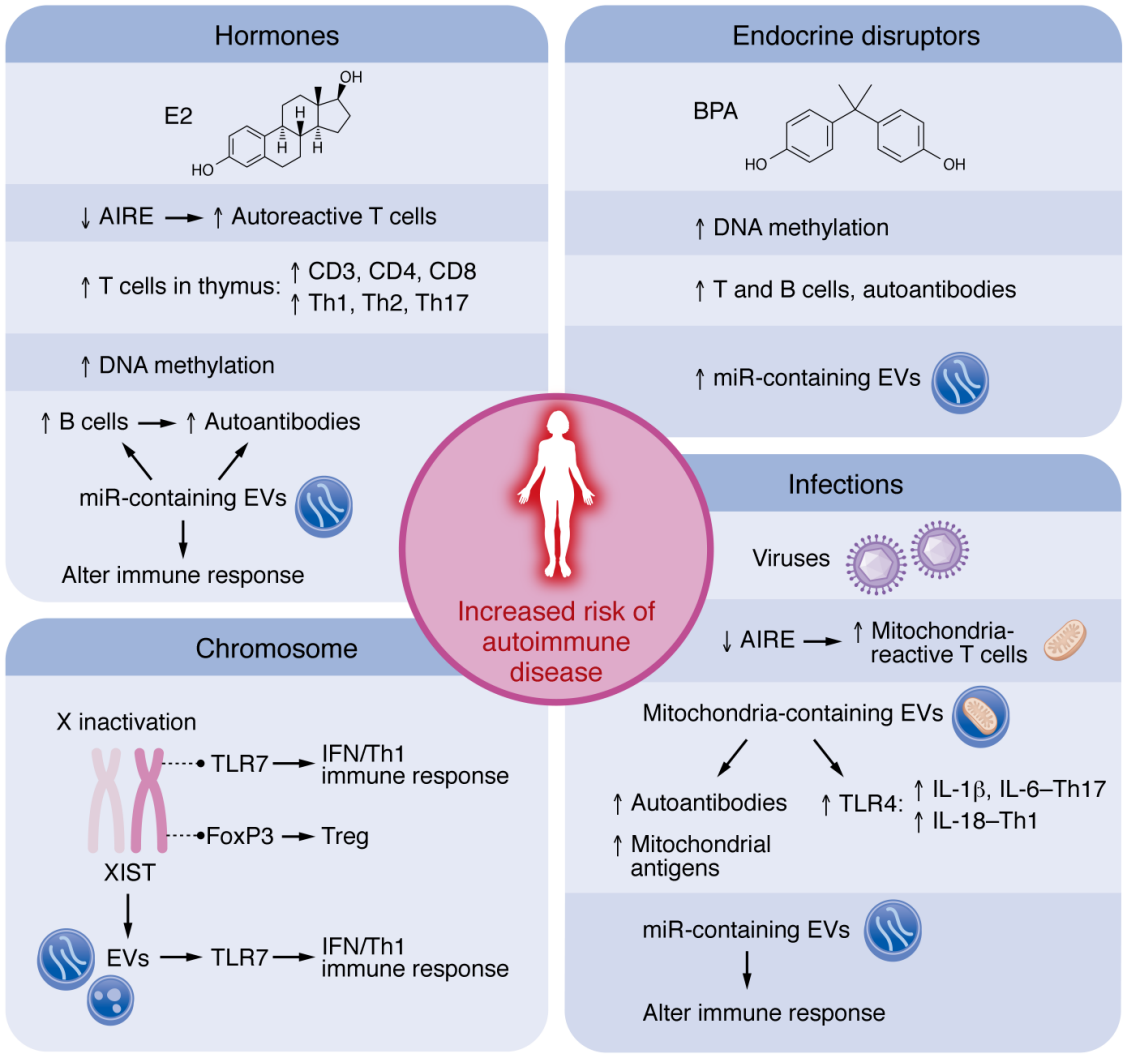
Potential mechanisms increasing the risk for autoimmune disease in women. Key factors that may increase the risk of developing autoimmune disease in females include genes on the X chromosome, sex hormones such as 17β-estradiol (E2), endocrine disruptors such as bisphenol A (BPA), and infections such as viruses. AIRE, autoimmune regulator gene; EVs, extracellular vesicles; FoxP3, forkhead box P3; mIR, microRNA; mito, mitochondria; XIST, X-inactive specific transcript.

One of the key immune factors that characterize autoimmune disease is the presence of autoantibodies. The effects of estrogens, and especially 17β-estradiol (E2/estrogen), are mediated primarily via estrogen receptor α (ERα) and ERβ, which are expressed in many immune cell types, including mast cells, macrophages, DCs, T cells, and B cells (29–31). Along with playing a classical role in genomic transition, ERs — which are also expressed on the surface of cells, including immune cells — can initiate rapid responses as part of lipid signaling rafts (reviewed in refs. 32, 33 ). Estrogen promotes activation and survival (34) and hypermutation and class switch recombination (35) in B cells, leading to higher antibody/autoantibody responses in females (36) (Figure 3). The effects of estrogen on antibody production in females are best illustrated following a viral infection. For example, women are reported to have higher antibody responses to the influenza vaccine compared with men regardless of age, dose of vaccine, or influenza strain (37). Similarly increased responses are observed in C57BL/6 and DBA/2 female mice after infection (38, 39). Exogenous estrogen administration increases autoantibody levels in both male and female mice in various mouse strains and animal models of autoimmune disease (reviewed in ref. 40).

### Sex hormones and autoreactive T cells.

Another key feature of autoimmune disease is the presence of autoreactive T cells. A critical protective feature of central tolerance is the autoimmune regulator gene (*AIRE*), which encodes a transcription factor that protects against self-reactivity by inducing expression of tissue-specific antigens that are normally not expressed in the thymus (41). Such tissue-specific antigens expressed by medullary thymic epithelial cells (mTECs) can be directly presented to developing T cells, or resident DCs in the thymus may take up these self-proteins and present them to T cells. If reactivity to self-antigen is too strong, mTECs or DCs provide signals that destroy the autoreactive T cells (41). Importantly, estrogen has been found to decrease AIRE expression, while androgen increases its expression (42, 43) (Figure 3), providing one possible explanation for the greater susceptibility of females to develop autoreactive T cells and autoimmune disease.

Additionally, estrogen is necessary for the development of T cells in the thymus (44). This may be why females have elevated T cell responses (i.e., CD3^+^, CD4^+^, and CD8^+^) and elevated Tregs compared with males (45) (Figure 3). However, the effect of estrogen on the type of CD4^+^ Th cell response to antigen varies by dose. Low-dose estrogen acting via ERα binds to the nuclear estrogen response element to promote IFN-γ and Th1-type immune responses in female C57BL/6 mice, as well as in male C57BL/6 mice that received orchidectomy and E2 supplementation (46–49). In contrast, estrogen at high concentrations promotes Th2-type immune responses associated with IL-4, IL-13, IL-33, and IL-10 production (40). Importantly, estrogen also promotes the development of Tregs that regulate antigen/autoantigen-specific Th responses in women and female C57BL/6 mice (50–53), suggesting that females should be good at regulating proinflammatory responses. However, the protective effect of estrogen goes awry in autoimmune diseases. Far less research has been conducted on the role of androgens on the immune response, but in general, androgens have been found to increase Th1 responses in males (54, 55).

### Estrogen and innate immunity.

Although antigen/autoantigen-specific immunity is mediated by T and B cells, innate immune cells such as mast cells, DCs, neutrophils, monocytes, and macrophages that initiate adaptive immune responses to environmental pathogens, toxins, or self-antigens can also mediate acute and chronic autoimmune pathology. Human and mouse mast cells, monocytes, and macrophages express ERα, ERβ, and the androgen receptor (30, 56). Androgen receptors are expressed at higher levels on macrophages in males than females (57). Estrogen via ERα has been shown to differentiate monocytes into inflammatory DCs, which present antigen to T cells and promote Th1 responses in female C57BL/6 mice and healthy human donors (58, 59). However, E2 activation of macrophages via ERα was also found to drive Th2 immune responses with increased GATA-3 and IL-4 in ovariectomized female C57BL/6 mice (60). The dose of estrogen seems to greatly affect monocytes and macrophages, with low doses promoting proinflammatory cytokines such as IL-1β, IL-6, and TNF, and high doses inhibiting these responses in humans and female C57BL/6 mice (61–63), similar to its effect on T cells. Estrogen has been found to downregulate certain innate immune pathways, such as TLR4 and the inflammasome are found on/in innate immune cells including mast cells and macrophages leading to a reduction in IL-1β and IL-6 (64–67). Another reason for the differential effects of estrogen is that ERα and ERβ exert opposite effects on the immune response (68–71), and their varying expression on immune cells may alter the effects of estrogen. Thus, estrogen appears to have a more complex effect on innate immune cells than on T and B cells (72); overall, however, sex steroids have profound effects on the immune response, providing at least part of the explanation for sex differences in the pathogenesis of autoimmune disease.

## The role of genes

A combination of genetic predisposition and environmental factors contributes to the development of autoimmune diseases (Figure 1), which are known to cluster in families and in individuals (i.e., individuals with one autoimmune disease are more likely to develop another autoimmune disease) (73). There is also a higher probability that family members without autoimmune disease will develop autoantibodies. The likelihood of developing similar autoantibodies relates directly to the sharing of human lymphocyte antigen (HLA) haplotypes with family members (74), and the probability is even greater if two haplotypes rather than one are shared. HLA haplotype, or the MHC in mice, is proposed to increase the prevalence of autoimmune disease by enhancing or altering self-antigen presentation in the periphery, resulting in increased autoreactive T cell activation (Figure 1). Most autoimmune diseases are thought to be polygenic, i.e., involving more than one gene, and many of the genes conferring susceptibility involve the immune response. Genetic predisposition to autoimmune disease can involve genes/genetic variants and noncoding microRNAs (miRs) or long noncoding RNAs (lncRNAs) (75).

### Role of X chromosome genes.

Increasing evidence indicates a role for the X chromosome in promoting autoimmune disease in females, as the number of X chromosomes in an individual is associated with an increased risk of developing an autoimmune disease (female 46XX, male 46XY) (19, 76, 77). To normalize the dose of gene expression in females (46XX), one X chromosome is randomly inactivated in each cell by X-inactive specific transcript (XIST). However, some genes (15%–23%) escape X chromosome inactivation, leading to a double dose of the encoded proteins, which can promote inflammation and autoimmune disease in females (Figure 3). Strikingly, females (47,XXX) or males (47,XXY) with an extra X chromosome are more commonly identified among individuals with Sjögren’s disease or SLE (78). Several immune genes (*FOXP3*, *IL2RG*, *TLR7*, *TLR8*, *CD40LG*, *BTK*, *CXCR3*) that are associated with increasing the likelihood of developing autoimmune disease in females are encoded on the X chromosome (77) (Figure 2). For example, the following are encoded on the X chromosome and found to be overexpressed in lymphocytes of females with SLE: the gene for forkhead box P3 (*FOXP3*), which increases Treg function; CD40 ligand (*CD40LG*), which allows T cells to activate B cells; Bruton tyrosine kinase (*BTK*), which is essential for the development and maturation of B cells; *TLR7*, which is a pattern recognition receptor that binds single-stranded RNA (ssRNA) and increases production of type I IFNs (IFN-α/β), resulting in elevated IFN-γ (resulting in elevated Th1 immune responses) and B cell activation; and chemokine (C-X-C) receptor 3 (*CXCR3*), which increases IFN-γ/Th1 immune cell responses to CXCL-9, CXCL-10, and CXCL-11 (45, 79–81). A gain-of-function *TLR7* variant that was identified in a child with severe lupus and then introduced into mice induced lupus-like features in a B cell–intrinsic manner independent of the formation of follicles or germinal centers (81). Evidence for a role of TLR7 in SLE was further strengthened by the finding that hyperactivation of TLR7 associated with early-onset SLE; the hyperactivation was attributed to mutations in genes that encode proteins required for proper control of TLR7 levels and function (82, 83). TLR7 has also been linked to the pathogenesis of other autoimmune diseases besides SLE, including type 1 diabetes and Sjögren’s disease, by increasing type I IFNs (25, 84, 85). These data indicate that TLR7 is a mediator of some autoimmune diseases, although all these genes could increase the risk of autoimmune disease in females.

Additionally, a large percentage of the genome encodes transcripts that are not translated into proteins, such as miRs and lncRNAs. miRs can be free floating in the cell, where they are produced or released to the local environment or circulation in extracellular vesicles (EVs) (85). miR content within EVs has been reported to be elevated in many autoimmune diseases and shown to have roles in promoting and/or regulating disease (75, 87–90). In one study, around 10% of the miRs in the human genome were located on the X chromosome (91), suggesting that their expression may be higher in females. The lncRNA XIST, which is necessary for X chromosome inactivation, was found in EVs released from apoptotic cells in culture after UV irradiation (92). Crawford et al. showed that XIST lncRNA from EVs was able to bind to and activate TLR7 and increase IFN-γ levels in culture, and that XIST was expressed more often in females (*n* = 12) with SLE than age-matched controls (*n* = 11) (92). These findings suggest that XIST could activate TLR7 in females in a sex-specific manner, promoting autoreactive antibodies and SLE (Figure 3). Evidence for this idea was further supported by Dou et al., who showed that expression of XIST in male C57BL/6 mice induced autoantibodies and exacerbated disease in a mouse model of SLE (93). Additional evidence of the importance of XIST in regulating TLR7 was recently reported by Huret et al., who prevented XIST inactivation in C57BL/6 mice, which resulted in elevated expression of TLR7, TLR8, TLR13, and CXCR3 on splenocytes, leading to elevated anti-DNA and anti-RNA serum antibodies and splenic TNF, IL-1β, and IL-6 levels in 1-year old mice (94). Thus, genes and noncoding transcripts found on the X chromosome may increase susceptibility to autoimmune disease in women.

### Insight provided by Four-Core Genotypes mouse model.

The Four-Core Genotypes (FCG) and similar mouse models (95) are useful for determining whether the causes of sex differences that are observed in phenotypes are due to hormonal effects, sex-chromosomal effects, or both. In FCG mice, the sex-determining region of the Y chromosome (*Sry*) is removed from the Y chromosome and provided through an independent transgene. *Sry* encodes the testis-determining factor, which initiates male sex determination. The FCG model produces four genotypes in which the sex characteristics are due to the presence or absence of *Sry* and independent of sex chromosomes, resulting in: XX with ovaries, XX with testes, XY with ovaries, or XY with testes. Comparison of the XX and XY mice with the same type of gonad (i.e., either containing *Sry* and testes or lacking *Sry* and containing ovaries) has led to discovery of phenotypes in which the complement of sex chromosomes causes sex differences. Comparison of mice with testes versus ovaries, with the same sex chromosomes, has led to discovery of phenotypes in which the presence or absence of *Sry* causes sex differences, including the effects of testicular versus ovarian secretions (95, 96). The models have been used to uncover sex chromosome contributions to sex differences in a wide variety of tissues and disease states, including the brain, heart, and immune system, as well as cardiovascular, autoimmune, and Alzheimer’s disease (95–98). In some cases, use of these models has led to the discovery of specific X or Y genes that protect from or exacerbate disease (97). Additionally, when sex chromosome and hormonal factors interact, they can reduce each other’s effects, which may not be discovered without the use of tools such as these.

## The role of environment

Although genetic factors are important in the development of autoimmune disease, twin studies indicate that environmental factors are a significant contributor (99–103). However, disentangling environmental from genetic contributions to autoimmune disease is complicated by epigenetic regulation of genes by the environment. Examples of environmental exposures associated with autoimmune diseases include infections, pesticides, solvents, endocrine-disrupting agents such as bisphenol A (BPA), occupational exposure to respirable particulates and fibers, and personal factors such as cigarette smoking history and diet (21, 104–107) (Figure 3).

### Role of epigenetics.

DNA methylation, histone modification, and regulation by miRs are important epigenetic mechanisms that influence the development of autoimmune disease (108, 109). Estrogen has been found to regulate DNA methylation in breast and endometrial cancers (110–112) and to enhance global hypomethylation of CD4^+^ T cells from patients with SLE, promoting disease (113) (Figure 3). Thus, estrogen-induced epigenetic regulation of gene expression could increase the susceptibility of women to autoimmune disease.

miRs are short (22 nucleotides), single-stranded, noncoding RNAs that form complementary base-pairs with the 3′ untranslated region of target mRNAs within the RNA-induced silencing complex (RISC) and block the function of protein-coding mRNAs (86). It has been reported that the human genome contains approximately 2,500 mature miRs that regulate approximately 60% of mRNAs (114). As mentioned above, the X chromosome has been estimated to regulate 10% of miRs (91). Additionally, miR transcription has been found to be regulated through ERα and ERβ in a tissue-specific and cell-dependent manner (86), producing so-called estrogen-related miRs. Estrogen-related miRs, such as miR-125 and miR-155, are thought to mediate the ability of estrogen to activate B cells to increase antibody/autoantibody production (86, 115) (Figure 3). Based on these findings, we would expect many miRs to differ by sex. Evidence for this includes a study by Dai et al. that reported sex differences in lupus-associated miRs in the NZB/WF1 mouse model of SLE (66), indicating their potential role in driving autoimmune disease in females. An avid area of research for many diseases, including autoimmune diseases, examines whether circulating EVs with specific miR content have the potential to be effective biomarkers of disease (116).

### Role of endocrine disruptors.

An important environmental factor that may influence sex differences in immune function is endocrine disrupting chemicals such as phenols (e.g., BPA, BPS), parabens, and phthalates, which can change immune function by altering binding of sex hormones to their receptors or sex hormone production (106, 109, 117, 118) (Figure 3). Endocrine-disrupting chemicals are now ubiquitous not only in the environment at large, but also in the environment of our animal models and cell culture experiments (e.g., there are endocrine-disrupting chemicals in culture media and leaching from warm plastic culture trays; and mice are housed in plastic cages with plastic water bottles and estrogenic compounds in their food and bedding). For example, BPA is a nonsteroidal xenoestrogen that exhibits 10^–4^ the activity of E2 and can inhibit the androgen receptor (119). Studies of the effect of BPA on immune cells in culture and animal models are abundant, revealing varied effects (reviewed in ref. 119). Some studies indicate that exposure to BPA or other endocrine disruptors can make disease worse, such as collagen-induced arthritis in male DBA/J mice (120), type 1 diabetes (reviewed in ref. 121), SLE in various models (122–124), and myocarditis in male and female BALB/c mice (106, 125). BPA is also able to disrupt DNA methylation (109, 126). Importantly, these effects are transgenerational (126, 127), and exposures continue after birth. Exposure to BPA has been found to lead to hypomethylation of CD4^+^ T cells in SLE and other autoimmune diseases compared with controls and to contribute to disease (126, 128), similar to the effects of estrogen. Because this is a new area of research there are only a few studies reporting the effects of endocrine disruptors on miRs (reviewed in refs. 86, 109), but with the high regulation of miRs by estrogen, endocrine disruptors may influence their production. However, more research is needed in animal models and human disease to confirm the role of endocrine disruptors in specific autoimmune diseases.

### Role of infection.

For many decades, viral and other infections have been suspected as “triggers” of autoimmune disease (90, 129), but mechanisms for how this could occur have been difficult to establish (130, 131). The COVID-19 pandemic brought viral infections back into the focus, as SARS-CoV-2 was found to increase myocarditis by at least 15-fold, from around 1–10 cases per 100,000 before COVID to 150 cases per 100,000 or more during the pandemic (132, 133). Additionally, several new diseases emerged from the pandemic that were reminiscent of autoimmune diseases, such as multisystem inflammatory syndrome in children (MIS-C) and long COVID in women (134–136).

Recent findings indicate that many viruses target mitochondria for a replicative advantage and subvert EV pathways to hide within EVs and evade the immune response, such as coxsackievirus, influenza, HIV, and SARS-CoV-2 (reviewed in refs. 137–142). In this process, EVs are created that contain mitochondria/mitochondrial and virus/viral components, altered miRs, and typical EV content (138, 143, 144). These EVs are expected to be highly immunogenic, because many of the constituents of the mitochondria within the EVs — e.g., mitochondrial cardiolipin, cytochrome *c*, and ATP — are known to activate TLR4 (145–149). Increased levels of extracellular mitochondria (likely housed in EVs) are observed in patients with rheumatic autoimmune diseases and are thought to contribute to disease (reviewed in ref. 150). Activation of innate immune cells by these mitochondrion-containing EVs may generate autoimmune responses against antinuclear cellular components, particularly antinuclear antibodies (ANAs), which are primary autoantigens in rheumatic autoimmune diseases (Figure 3). Additionally, antimitochondrial antibodies (e.g., antibodies that target cardiolipin, mitofusin 1, mitochondrial DNA, or mitochondrial RNA) are commonly found in patients with rheumatic autoimmune diseases such as rheumatoid arthritis, SLE, and antiphospholipid syndrome (150). Elevated levels of EVs containing mitochondria are positively associated with increased SLE disease activity, proinflammatory cytokines, and anti-dsDNA antibodies, suggesting that EVs with mitochondrial components may be involved in disease pathogenesis (150, 151). Elevated mitochondrial levels in red blood cells have been found to increase IFN responses in patients with SLE (152, 153). TLR4/inflammasome activation by mitochondrial components can lead to elevated IFNs via IL-18, which was initially called IFN-inducing factor, in addition to traditional IFN pathways such as MAVS/STING (154). These findings suggest that damage to mitochondria from viral infections, drugs, or toxins may increase autoimmune responses and serve as a common mechanism in the pathogenesis of several autoimmune diseases.

The question is whether viral infection or other causes of damage to mitochondria can increase susceptibility to autoimmune disease in females. Mitochondrial energetics (that is, morphology, biogenesis, respiration, reactive oxygen species, etc.) are known to differ by sex (155, 156). For example, mitochondria from the hearts of female rodents and human cardiomyocytes in culture are known to have greater efficiency, fatty acid utilization during exercise, and calcium retention; whereas males have more mitochondrial content, reactive oxygen species production, and a higher calcium uptake rate (28, 157). A major transcriptional regulator of mitochondrial genes and function is ER-related receptor α (ERRα) (158, 159), which, although it does not have estrogen as a ligand, is associated with sex differences in mitochondrial function in a number of animal models (137, 160–162). PPARγ coactivator 1α (PGC1α), which is a known master regulator of mitochondrial function, is a cofactor for ERRα transcription (163). Thus, it would be expected that interference of mitochondrial function by viruses or other pathogens or toxins may differ by sex, and the release of EVs with mitochondrial content may promote sex-specific immune responses, increasing the risk of autoimmune disease in females (Figure 3). Future studies are needed to confirm this hypothesis.

## Pediatric autoimmune diseases

Generally, in autoimmune diseases that occur during both childhood and adulthood — including SLE, Sjögren’s disease, systemic sclerosis, multiple sclerosis, celiac disease, and autoimmune thyroid disease — sex differences are less pronounced in children, with a more modest female predominance (164–166). For example, female-to-male ratios of Sjögren’s disease in adults are as high as 21:1 in the 19- to 36-year age group and over 14:1 in all adults, but 6:1 in the pediatric age range (166). For autoimmune diseases more common in children, sex differences are relatively less female-skewed or more variable. For example, type 1 diabetes occurrence is nearly equal in males and females, perhaps with slight male predominance; while juvenile idiopathic arthritis (JIA) is more common in females (2:1 to 3:1), except for certain subtypes, such as systemic JIA (no sex difference) and enthesitis-related arthritis (slight male predominance) (164). While hormones change in a sex-specific manner during adolescence, sex differences in some autoimmune diseases occur during earlier childhood, when hormones are not sex dependent, suggesting that non-hormone-associated drivers of sex differences exist. More research is needed to better understand those factors.

## Summary

Most adult autoimmune diseases, especially those with the highest prevalence, occur more often in women than men. Decades of research provide a strong basis for the role of estrogen in promoting autoreactive T and B cells, leading to increased autoantibodies and clinical disease. However, research on the role that sex hormones and estrogen play in innate immune cells has generated highly variable result. Genes, including genes on the X chromosome, contribute to sex differences in autoimmune diseases, but environment and epigenetics are also a major contributor. Recent and rapidly evolving areas of research include the role of hormone-dependent miRs and EVs on promoting and/or protecting from autoimmune disease. The COVID-19 pandemic provided new insight into how viruses may alter miR and EV content to promote autoimmune diseases, reviving an old hypothesis.

## Gaps

Large advances in understanding have been made to identify the potential mechanisms leading to a higher prevalence of autoimmune disease in women. The 2015 requirement by NIH to report sex differences in cell, animal, and human research has led to an increase in reporting. However, a large quantity of past publications did not report the sex of the animal or cell type, and the experiments have not been repeated to determine whether sex differences exist. An often overlooked issue that affects interpretation of sex differences in the immune response is the presence or absence of mast cells in mouse strains. Mouse strains with many mast cells such as BALB/c mice develop predominant Th2 immune responses to antigens/autoantigens, while mouse strains with few mast cells such as C57BL/6 mice develop predominant Th1 immune responses. Thus, there is a need to report sex differences in mouse models in the context of mouse strain. Additionally, few studies exist on the effects of androgen receptor signaling in females and males. More research is also needed on the effect of endocrine disruptors on physiology and immune responses to self-antigens according to sex. Almost all studies that examine the role of sex hormones and endocrine disruptors do not control for the exposure of exogenous estrogens in the bedding, food, and culture media supplements, nor do they control for endocrine-disrupting agents such as plastics (i.e., BPA, BPF) in culture dishes, media, caging, and water. Another important gap is that there are no recent epidemiologic studies that report the prevalence of all autoimmune diseases in the US, or the prevalence of many individual autoimmune diseases along with their sex ratios. These data are needed to better understand sex differences in autoimmune disease and to stimulate research into understanding the mechanisms.

## Conclusions

Recent evidence provides an increasingly clear understanding of the role of sex hormones, genes, and environmental factors in promoting inflammation in females that leads to autoimmune disease. However, many gaps and questions remain. More research is needed to better understand how hormones affect physiology by organ and tissue and their effect on the immune response in models of autoimmune disease and in patients. Exciting new areas of investigation include sex differences in the epigenetic regulation of genes/RNA by miRs carried in EVs, which may serve as novel biomarkers, therapeutic targets, and/or potential therapies for autoimmune diseases that disproportionately affect women.

## Version 1 09/17/2024

Electronic publication
